# Reviewer Acknowledgements 2015

**DOI:** 10.1186/s13071-016-1422-8

**Published:** 2016-03-08

**Authors:** Chris Arme

**Affiliations:** Huxley Building, Keele University, Keele, ST5 5BG UK

## Abstract

*Parasites & Vectors* would like to thank everyone who reviewed for the journal in 2015.

**Rabindra Abeyasinghe**

Philippines

**Nicole Achee**

United States

**Mulugeta Aemero**

Ethiopia

**Yaw Afrane**

Ghana

**Philip Agnew**

France

**Lelo Agola**

Tanzania

**Leopoldina Aguirre Macedo**

Mexico

**Fola Agusto**

United States

**Arunee Ahantarig**

Thailand

**Hirotsugu Aiga**

Japan

**Amir Akhavan**

Iran

**Munir Aktas**

Turkey

**Mohammad Shafiul Alam**

Bangladesh

**Pedro Alarcón-Elbal**

Spain

**Pilar Alberdi**

Spain

**Abebe Alemu**

Ethiopia

**Kaio Alevi**

Brazil

**Neal Alexander**

United Kingdom

**Carlos Eduardo Almeida**

Brazil

**Hesham Al-Mekhlafi**

Malaysia

**Lionel Almeras**

France

**Salman Al-Shami**

Saudi Arabia

**Adam H. Al-Shamiri**

Yemen

**Bulent Alten**

Turkey

**Barry Alto**

United States

**Cosme Alvarado-Esquivel**

Mexico

**Cristian Alvarez Rojas**

Australia

**John Andersen**

United States

**Martin Andersson**

Sweden

**Andrey Andrade**

Brazil

**José Dilermando Andrade Filho**

Brazil

**Valter Andrade-Neto**

Brazil

**Renato Andreotti**

Brazil

**Samer Angelone-Alasaad**

Spain

**Giada Annoscia**

Italy

**Desiderato Annoscia**

Italy

**Brendan Ansell**

Australia

**Christophe Antonio-Nkondjio**

Cameroon

**Charles Apperson**

United States

**Jackson Araujo**

Brazil

**Márcio Araújo**

Brazil

**Minerva Arce-Fonseca**

Mexico

**Peter Armbruster**

United States

**Chris Arme**

United Kingdom

**Veronica Armijos**

United States

**Philip Armstrong**

United States

**Muhammad Ashfaq**

Canada

**Marek Asman**

Poland

**Katherine Atkins**

United Kingdom

**Stephen Atkinson**

United States

**Carter Atkinson**

United States

**Márcia Attias**

Brazil

**Stephen Attwood**

China

**Denis Augot**

France

**Andre Bafica**

Brazil

**Anna Bajer**

Poland

**Valdir Balbino**

Brazil

**Dan Baldassarre**

United States

**Rania Baleela**

United States

**Allison Bancroft**

United Kingdom

**Claudio Bandi**

Italy

**Gad Baneth**

Israel

**Jay Bara**

United States

**Ricardo Andrade Barata**

Brazil

**Alan Barbour**

United States

**Marcelo Barcinski**

Brazil

**Beatrice Barda**

Switzerland

**Vanessa Bardella**

Brazil

**Maria Dolores Bargues**

Spain

**Steve Barker**

Australia

**Don Barnard**

United States

**Joel Barratt**

Australia

**Roberto Barrera**

United States

**Jean-Mathieu Bart**

Spain

**Bruno Bassano**

Italy

**Paul Bates**

United Kingdom

**Matthew Baylis**

United Kingdom

**Norbert Becker**

Germany

**Nigel Beebe**

Australia

**Mike Begon**

United Kingdom

**Jerzy Behnke**

United Kingdom

**Jasminca Behrmann**

Germany

**John Beier**

United States

**Jean-Nicolas Beisel**

France

**Pablo Beldomenico**

Argentina

**Carlota Belisário**

Brazil

**Diana Bell**

United Kingdom

**Silvana Belo**

Portugal

**John Paul Benante**

United States

**Marlene Benchimol**

Brazil

**Daniel Benesh**

United States

**Kelly Bennett**

United Kingdom

**Staffan Bensch**

Sweden

**Robert Bergquist**

Sweden

**Scott Bernhardt**

United States

**Rasa Bernotiene**

Lithuania

**Colin Berry**

United Kingdom

**Esmael Besufikad**

Ethiopia

**Martha Betson**

United Kingdom

**Frederic Beugnet**

France

**Tapan Bhattacharyya**

United Kingdom

**Peter Billingsley**

United States

**Oliver Billker**

United Kingdom

**Hadush Birhanu**

Belgium

**Fadi Bittar**

France

**Samuel Black**

United States

**William Black IV**

United States

**Jenefer Blackwell**

Australia

**David Blair**

Australia

**Damer Blake**

United Kingdom

**Isobel Blake**

United Kingdom

**Mariana Boadella**

Spain

**Marlean Boelaert**

Netherlands

**Mariana Boité**

Brazil

**Bethany Bolling**

United States

**Mark Booth**

United Kingdom

**Michael Boots**

United Kingdom

**Fred Bordes**

France

**Robert Bos**

Switzerland

**Jérémy Bouyer**

Senegal

**Maha Bouzid**

United Kingdom

**Dwight Bowman**

United States

**Leigh Bowman**

United Kingdom

**Uffe Braae**

Denmark

**Laura Braden**

Canada

**Janette Bradley**

United Kingdom

**Oliver Brady**

United Kingdom

**Erika Braga**

Brazil

**Reginaldo Brazil**

Brazil

**Edward Breitschwerdt**

United States

**Alberto Bresciani**

Italy

**Emanuele Brianti**

Italy

**William Brieger**

United States

**Jory Brinkerhoff**

United States

**Andrew Briscoe**

United Kingdom

**Dustin Brisson**

United States

**Basil Brooke**

South Africa

**Simon Brooker**

United Kingdom

**Peter Brophy**

United Kingdom

**Robert Brown**

United States

**Bryan Brown**

United States

**Kurt Buchmann**

Denmark

**Amy Buck**

United Kingdom

**Alicja Buczek**

Poland

**Lilian Bueno**

Brazil

**Ruben Bueno-Mari**

Spain

**Hugo Bugoro**

Solomon Islands

**Nancy Bunbury**

Seychelles

**Stewart Burgess**

United Kingdom

**Alejandro Cabezas-Cruz**

France

**Jo Cable**

United Kingdom

**Beatris Cademartori**

Brazil

**Marie-Christine Cadiergues**

France

**Claudia Cafarchia**

Italy

**Francisco Fernando Calvo**

Belgium

**Mattia Calzolari**

Italy

**Vitaliano Cama**

United States

**Mary Cameron**

United Kingdom

**Corey L. Campbell**

United States

**Bronwyn Campbell**

Australia

**Lindsay Campbell**

United States

**Jorge Cano**

United Kingdom

**Cinzia Cantacessi**

Australia

**Jun Cao**

China

**Gioia Capelli**

Italy

**Helene Carabin**

United States

**Ring Carde**

United States

**Luis Cardoso**

Portugal

**Ryan Carnegie**

United States

**Ana Carolina Carneiro**

Brazil

**Yannick Caron**

Belgium

**Simon Carpenter**

United Kingdom

**Hernán Carrasco**

Venezuela

**Moacir Carretta**

Brazil

**Mark Carrington**

United Kingdom

**Rudi Cassini**

Italy

**Ricardo Castillo**

United States

**Socrates Cavalcanti**

Brazil

**Serena Cavallero**

Italy

**Giuliano Cecchi**

Ethiopia

**Neveu Cedric**

France

**Frank Cezilly**

France

**Dave Chadee**

Trinidad and Tobago

**Rachel Chalmers**

United Kingdom

**Ricky Chan**

United States

**Kshitij Chandel**

India

**Kwang Poo Chang**

United States

**David Chapman**

United States

**Lloyd Chapman**

United Kingdom

**Johannes Charlier**

Belgium

**Farhana Chaudhry**

Pakistan

**Robert Cheke**

United Kingdom

**Xiao-Guang Chen**

China

**Jia-Xu Chen**

China

**Qijun Chen**

China

**Daixiong Chen**

China

**Xiao-Ping Chen**

China

**Changde Cheng**

United States

**Guofeng Cheng**

China

**Neil Chilton**

United States

**Jan Chirico**

Sweden

**Bruno Chomel**

United States

**Valerie Choumet**

France

**Philippe Christe**

Switzerland

**Caroline Chua**

Malaysia

**Tock Chua**

Malaysia

**Young-Bae Chung**

Republic of Korea

**David Civitello**

United States

**Graham Clark**

United Kingdom

**Theodore Clark**

United States

**Joel Coats**

United States

**Maureen Coetzee**

South Africa

**Luc Coffeng**

Netherlands

**Gerald Coles**

United Kingdom

**Doug Colwell**

Canada

**Gary Conboy**

Canada

**Jan Conn**

United States

**Franz Josef Conraths**

Germany

**Michael Conway**

United States

**Vincent Corbel**

Benin

**Anthony Cornel**

United States

**Sofia Cortes**

Portugal

**Carlos Costa**

Brazil

**Jane Costa**

Brazil

**Jaime Costales**

Ecuador

**Mark Costello**

New Zealand

**James Cotton**

United Kingdom

**Jean T. Coulibaly**

France

**Jeroen Coumou**

Netherlands

**Jose Coura**

Brazil

**Janelle Couret**

United States

**Robert Cowie**

United States

**Douglas Craig**

Canada

**Lee Crainey**

United States

**Robert Creed**

United States

**Thomas Cribb**

Australia

**Giuseppe Cringoli**

Italy

**Andrea Crisanti**

United Kingdom

**Dac Crossley**

United States

**Ron Crump**

United Kingdom

**Jing Cui**

China

**Narcisa Cunha-E-Silva**

Brazil

**Elisa Cupolillo**

Brazil

**Douglas Currie**

Canada

**Rachel Curtis**

United States

**Cristina Cutillas**

Spain

**Sally Cutler**

United Kingdom

**Andre Cutolo**

Brazil

**Aleksandro Da Silva**

Brazil

**Ary Gomes Da Silva**

Brazil

**João Santana da Silva**

Brazil

**Itabajara da Silva Vaz Jr**

Brazil

**Fredrick Dahlgren**

United States

**Jianfeng Dai**

China

**John Dalton**

United Kingdom

**Stefano D'Amelio**

Italy

**Gianluca D'Amico**

Romania

**Renita Danabalan**

Germany

**Filipe Dantas-Torres**

Brazil

**Frédéric Darriet**

France

**Paban Dash**

India

**Sunando Datta**

India

**Arwid Daugschies**

Germany

**Hans Dautel**

Germany

**Rebecca Davidson**

Norway

**Tim Day**

United States

**Ujjwal De**

India

**Jose De La Fuente**

Spain

**Claudio De Liberato**

Italy

**Wanderley De Souza**

Brazil

**Alex Debrah**

Ghana

**Paron Dekumyoy**

Thailand

**Leen Delang**

Belgium

**Helene Delatte**

France

**Vincent Delespaux**

Belgium

**Alessandra Della Torre**

Italy

**Paul Denny**

United Kingdom

**Lindsay Dent**

Austria

**Jerome Depaquit**

France

**Peter Deplazes**

Switzerland

**Marcel Deponte**

Germany

**Marketa Derdakova**

Slovakia

**Shaun Dergousoff**

Canada

**Kebede Deribe**

Ethiopia

**Nicole Desbois**

France

**Ranadhir Dey**

United States

**Sunil Dhiman**

India

**Angela Di Cesare**

Italy

**Abdoulaye Diabate**

Burkina Faso

**Anastasia Diakou**

Greece

**Diawo Diallo**

Senegal

**Fernando Dias**

Brazil

**Carl Dick**

United States

**Manlio Dicristina**

Italy

**Muriel Dietrich**

Reunion

**Colette Dissous**

France

**Armel Djenontin**

Benin

**Rosseau Djouaka**

Benin

**Henryka Długońska**

Poland

**Gerhard Dobler**

Germany

**Andrew Dobson**

United Kingdom

**Stephen Dobson**

United States

**Roberto Docampo**

United States

**Michael J. Doenhoff**

United Kingdom

**Ana Domingos**

Spain

**Martin Donnelly**

United Kingdom

**Pierre Dorny**

Belgium

**Gilles Dreyfuss**

France

**Patrick Driguez**

Australia

**Michael Dryden**

United States

**Matthew Dryden**

United Kingdom

**Ai-Fang Du**

China

**Mariana Duarte**

Brazil

**Jitender Dubey**

United States

**Jean Dubremetz**

France

**Luc Duchateau**

Belgium

**Giles Duffield**

United States

**Dagne Duguma**

United States

**Remy Durand**

France

**Georg Duscher**

Austria

**Isabelle Dusfour**

France

**Eran Dvir**

South Africa

**Gregory Ebel**

United States

**Andrea Egizi**

United States

**M. A. Ehsan**

Bangladesh

**Rebecca Eisen**

United States

**Uwem Ekpo**

Nigeria

**Ayman El-Badry**

Egypt

**Naglaa El-Lakkany**

Egypt

**Ivo Elliot**

United Kingdom

**John Ellis**

Australia

**Dia-Eldin Elnaiem**

United States

**Monica Embers**

United States

**Aidan Emery**

United Kingdom

**Nancy Endersby**

Australia

**David Engman**

United States

**Marina Ereveeva**

United States

**Kamil Erguler**

Cyprus

**Koray Ergunay**

Turkey

**Ozge Erisoz Kasap**

Turkey

**Roger Eritja**

Spain

**Nargys Es-Sette**

Morocco

**Johan Esterhuizen**

Uganda

**Maria Esteve-Gassent**

United States

**Agustin Estrada-Peña**

Spain

**Roger Evans**

United Kingdom

**Roy Faiman**

Israel

**Chia-Kwung Fan**

Taiwan Republic of China

**Fang Fang**

China

**Rui Fang**

China

**Chafika Faraj**

Morocco

**Ary Faraji**

United States

**Fernanda Faria**

Brazil

**Rodolfo Andrés Farlora Zapata**

Chile

**Mark Fast**

Canada

**Christina Faust**

United States

**Ronald Fayer**

United States

**Nina Fefferman**

United States

**Xin-Gang Feng**

China

**Yaoyu Feng**

China

**Tsegaw Fentie**

Ethiopia

**Heather Ferguson**

United Kingdom

**Martina Ferraguti**

Spain

**Marcelo Ferreira**

Brazil

**Elizabeth Ferrer**

Venezuela

**Ezio Ferroglio**

Italy

**Eric Fèvre**

United Kingdom

**Elisabeth Fichet-Calvet**

Germany

**Luiz Tadeu Figueiredo**

Brazil

**Luciana Figueredo**

Brazil

**Julio V. Figueroa**

Mexico

**Jordi Figuerola**

Spain

**Paul Fine**

United Kingdom

**Katja Fischer**

Australia

**Peter Fischer**

United States

**Durland Fish**

United States

**Ana Flisser**

Mexico

**Adriana Flores**

Mexico

**Fernando Flores-Pérez**

Mexico

**Robin Flynn**

United Kingdom

**Gabor Foldvari**

Hungary

**Desmond Foley**

United States

**Cristina Fonseca**

Brazil

**Dina Fonseca**

United States

**Dina Fonseca**

United States

**Albin Fontaine**

France

**Fabienne Forton**

Belgium

**Woodridge Foster**

United States

**Antonio Frangipane Di Regalbono**

Italy

**Mark Freeman**

Malaysia

**Simone Freitas**

Brazil

**Michael French**

United Kingdom

**Roger Frutos**

France

**Bao-Quan Fu**

China

**Hans-Peter Fuehrer**

Austria

**Màrius Fuentes**

Spain

**Ricardo Fujiwara**

Brazil

**Jenna Fyfe**

United Kingdom

**Endalamaw Gadisa**

Ethiopia

**Gabriella Gaglio**

Italy

**Francis Gakuya**

Kenya

**Eunice Galati**

Brazil

**Fredy Galvis Ovallos**

Brazil

**Manoj Gambhir**

Australia

**Eduardo Garcia-Zepeda**

Mexico

**Mario Garrido**

Spain

**Lindsey Garver**

United States

**Katie Gass**

United States

**Robin Gasser**

Australia

**Charles Gauci**

Australia

**Michael Gaunt**

United Kingdom

**Alessia Gazzonis**

Italy

**Timothy Geary**

Canada

**Stefan Geiger**

Brazil

**Peter Geldhof**

Belgium

**Claudio Genchi**

Italy

**Solange Gennari**

Brazil

**Mónica Germano**

Argentina

**Dirk Geysen**

Belgium

**Paul Giacomin**

Australia

**Annunziata Giangaspero**

Italy

**Alessio Giannelli**

Italy

**Gabriella Gibson**

United Kingdom

**Wendy Gibson**

United Kingdom

**Nelly Gidaszewski**

France

**John Gimnig**

United States

**Michael Ginger**

United Kingdom

**Yvette Girard**

United States

**Pedro Giulianini**

Italy

**Olivier Glaizot**

Switzerland

**Robert Glaser**

United States

**Travis Glenn**

China

**Andea Gloria-Soria**

United States

**Katey Glunt**

United States

**Geoffrey Gobert**

Australia

**Alexander Gofton**

Australia

**Nick Golding**

United Kingdom

**Daniel Gomes**

Brazil

**Maria Gomes-Solecki**

United States

**Jorge Gomez-Marin**

Colombia

**Andres Gomez-Palacio**

Colombia

**Suênia Gonçalvez**

Brazil

**Clara González**

Colombia

**Camila Gonzalez Rosas**

Colombia

**Mary Gonzatti**

Venezuela

**Catherine Gordon**

Australia

**David Gorla**

Argentina

**Nicole Gottenker**

United States

**Anouk Gouvras**

United Kingdom

**Nicodem James Govella**

Tanzania

**M Govindarajan**

India

**Dennis Grab**

United States

**Daniel Grabner**

Germany

**Luigi Gradoni**

Italy

**Thirssa H. Grando**

Brazil

**Erik Granquist**

Norway

**Britton Grasperge**

United States

**Jeremy Gray**

Ireland

**Marta Grech**

Argentina

**Ryan Gregory**

Canada

**Megan Greischar**

Canada

**Christoph G. Grevelding**

Germany

**Parwinder Grewal**

United States

**Christine Griffin**

Ireland

**Linda Grigoraki**

Greece

**Mario Grijalva**

United States

**Jack Grimes**

United Kingdom

**Alex Grinberg**

New Zealand

**Edmundo Grisard**

Brazil

**Maureen Groer**

United States

**Arthur Gruber**

Brazil

**Sarah Guagliardo**

United States

**Gui-Quan Guan**

China

**Simon Gubbins**

United Kingdom

**Herbert Guedes**

Brazil

**Patrick Guerin**

Switzerland

**Felipe Guhl**

Colombia

**Favia Guido**

Italy

**Jacques Guillot**

France

**Nayana Gunathilaka**

Sri Lanka

**Filiz Gunay**

Turkey

**Shivali Gupta**

United States

**David Gurarie**

United States

**Rodrigo Gurgel-Goncalves**

Brazil

**Ricardo Gürtler**

Argentina

**Helen Guyatt**

France

**Karen Haag**

Brazil

**Silvia Haase**

Australia

**Andrew Haddow**

United States

**Stephen Hadfield**

United Kingdom

**Carina Hall**

United States

**Sascha Hallett**

United States

**Jo Halliday**

United Kingdom

**Omar Hamarsheh**

Palestinian Territory Occupied

**Patrick Hamilton**

United Kingdom

**Xiu-Min Han**

China

**Ahmad Hanafi-Bojd**

Iran

**Kurt Hanevik**

Norway

**Limb Hapiara**

Australia

**Ralph Harbach**

United Kingdom

**David Harrington**

Denmark

**Laura Harrington**

United States

**Leslie Harrison**

United Kingdom

**Bruce Harrison**

United States

**Lara Harrup**

United Kingdom

**Shimon Harrus**

Israel

**Mo'awia Hassan**

Sudan

**John Hawdon**

United States

**Frances Hawkes**

United Kingdom

**Hadas Hawlena**

Israel

**Allen Heath**

New Zealand

**Helena Helmby**

United Kingdom

**Andrew Hemphill**

Switzerland

**Anna Henningsson**

Sweden

**Conny Herholz**

Switzerland

**Luis Hernandez**

United Kingdom

**Geoff Hide**

United Kingdom

**Yousif Himeidan**

Sudan

**Joseph Hinnebusch**

United States

**Alexandra Hiscox**

Netherlands

**Mary Hodges**

Kenya

**Jane Hodgkinson**

United Kingdom

**Adnan Hodžić**

Austria

**Achim Hoerauf**

Germany

**Johan Höglund**

Sweden

**Deirdre Hollingsworth**

United Kingdom

**Deborah Holt**

Australia

**Sung-Jong Hong**

Republic of Korea

**David Hoole**

United Kingdom

**Martin Horn**

Czech Republic

**Sándor Hornok**

Hungary

**Gabriele Hrckova**

Slovakia

**Larry Hribar**

United States

**Wei Hu**

China

**Min Hu**

China

**Victor Hu**

United Kingdom

**Xuemei Hu**

China

**Yan Huang**

China

**Yiau-Min Huang**

United States

**Si-Yang Huang**

China

**Yan-Jang Huang**

United States

**Leon Eklund Hugo**

Australia

**Jean-Pierre Hugot**

France

**Sun Huh**

Republic of Korea

**Nguyen Manh Hung**

China

**Peter Hunt**

Australia

**Hilary Hurd**

United Kingdom

**Ana Hurtado**

Spain

**Mae Huynh**

United States

**Athanasios Iatropoulos**

Greece

**Roberta Iatta**

Italy

**Adolfo Ibanez-Justicia**

Netherlands

**Carlos Ibarra-Cerdeña**

Mexico

**Adriana Ibelli**

Brazil

**Ralf Ignatius**

Germany

**Hideo Imamura**

Belgium

**Juliana Inoue**

Brazil

**Furhan Iqbal**

Pakistan

**Seth Irish**

United States

**Peter Irwin**

Australia

**Allison Isaacs**

United Kingdom

**Mats Isaksson**

Sweden

**Akira Ito**

Japan

**Al Ivens**

United Kingdom

**Abdul Jabbar**

Australia

**Maxime Jacquet**

Switzerland

**Thomas Jaenson**

Sweden

**Dragana Jankovic**

United States

**Cassie Jansen**

Australia

**Ana Maria Jansen**

Brazil

**Fakhri Jeddi**

France

**Henrik Jensen**

Denmark

**Aaron Jex**

Australia

**Wanzhong Jia**

China

**Anja Joachim**

Austria

**Magnus Johansson**

Sweden

**Brian Johnson**

Australia

**Doug Jones**

United States

**Malcolm Jones**

Australia

**Frans Jongejan**

Netherlands

**Luiz Juliano**

Brazil

**Tae Sung Jung**

Republic of Korea

**Silvia Justi**

Brazil

**Martin Kalbe**

Germany

**Joshua Kamani**

Nigeria

**Joseph Kamau**

Kenya

**Basile Kamgang**

France

**Ayubo Kampango**

Mozambique

**Helge Kampen**

Germany

**Panagiotis Karanis**

Germany

**Grzegorz Karbowiak**

Poland

**Mohammad Hossein Karimi**

Iran

**Egil Karlsbakk**

Norway

**Hirotomo Kato**

Japan

**Naoko Kato-Hayashi**

Japan

**Frank Katzer**

United Kingdom

**Hitoshi Kawada**

Japan

**Paul Kaye**

United Kingdom

**Maria Kazimirova**

Slovakia

**Jennifer Keiser**

Switzerland

**Steven Kelly**

United Kingdom

**Louise Kelly-Hope**

United Kingdom

**Malcolm Kennedy**

United Kingdom

**Derek Kennedy**

Australia

**Ambaye Kenubih**

Ethiopia

**Fiona Kenyon**

United Kingdom

**Stella Kepha**

Kenya

**Naveed Khan**

Pakistan

**Maxim Khasnatinov**

Russian Federation

**Nobuhide Kido**

Japan

**Christian Kiffner**

Germany

**Gerry Killeen**

Tanzania

**Dong Min Kim**

Taiwan Republic of China

**Phillipa King**

United Kingdom

**Carsten Kirkeby**

Denmark

**Shinichi Kitamura**

Japan

**Jovin Kitau**

Tanzania

**Sawanyawisuth Kittisak**

Taiwan Republic of China

**Sonia Kjos**

United States

**Claudia Klein**

Canada

**Terry Klein**

United States

**Daniel Kline**

United States

**Stephen Klotz**

United States

**Stefanie Knopp**

Switzerland

**Kevin Kobylinski**

Thailand

**Alicia Kocisova**

Slovakia

**Lizette Koekemoer**

South Africa

**Jacob Koella**

France

**Constantianus J.M. Koenraadt**

Netherlands

**Ismail Koltas**

Turkey

**Nicholas Komar**

United States

**Yoon Kong**

Republic of Korea

**Pasi Korhonen**

Australia

**Aneta Kostadinova**

Czech Republic

**Linda Kothera**

United States

**Michalis Kotsyfakis**

Czech Republic

**Benjamin Koudou**

United Kingdom

**Artemis Koukounari**

United Kingdom

**Moritz Kraemer**

United Kingdom

**E Krafsur**

United States

**Lena Krayter**

Germany

**Michael Kristensen**

Denmark

**Jürgen Krücken**

Germany

**Szu-Cheng Kuo**

Taiwan Republic of China

**Francesco La Torre**

Italy

**Norma Labarthe**

Brazil

**Pierrick Labbé**

France

**Lisa Labbé Sandelin**

Sweden

**Marcelo Labruna**

Brazil

**Shannon Ladeau**

United States

**Thewarach Laha**

Thailand

**De-Hua Lai**

China

**Iain Lake**

United Kingdom

**Patrick Lammie**

United States

**Sai-Gek Lam-Phua**

Singapore

**Humberto Lanz-Mendoza**

Mexico

**Gabriel Laporta**

Brazil

**Michael Lappin**

United States

**Abdalla Latif**

South Africa

**Maria Stefania Latrofa**

Italy

**Audrey Lau**

United States

**Fabrice Laurent**

France

**Juan Lauthier**

Brazil

**Andrea Lawrence**

Australia

**Scott Lawton**

United Kingdom

**Phillip Lawyer**

United States

**Walter Leal**

United States

**Andy Leisewitz**

South Africa

**Paul Leisnham**

United States

**Manigandan Lejeune**

Canada

**Francisco Lemos**

Brazil

**Laetitia Lempereur**

United Kingdom

**Matthias Lendner**

Germany

**Audrey Lenhart**

United States

**Samson Leta**

Ethiopia

**Michael Levin**

United States

**Michael Levy**

United States

**Michael Lewis**

United Kingdom

**Xiangrui Li**

China

**An-Xing Li**

China

**Wei Li**

China

**Youquan Li**

China

**Guodong Liang**

China

**Paulo Liedo**

United States

**Lisette Lieshout**

Netherlands

**Marshall Lightowlers**

Australia

**Jiaojiao Lin**

China

**David Lindsay**

United States

**Jo Lines**

United Kingdom

**Yvonne-Marie Linton**

United States

**Susan Little**

United States

**Guang-Yuan Liu**

China

**Guo-Hua Liu**

China

**Jingze Liu**

China

**Jun Liu**

United States

**Jianzhu Liu**

China

**Mingyuan Liu**

China

**Quan Liu**

China

**Xinjian Liu**

China

**Zhijie Liu**

China

**Zhi Long Liu**

China

**Martin Llewellyn**

United Kingdom

**David Lloyd**

United Kingdom

**Mike Lo**

United States

**Jose Loaiza**

Panama

**Melissa Lodoen**

United States

**Carlos Logullo**

Brazil

**Eric Loker**

United States

**Fabrizio Lombardo**

Italy

**Gabriella Lombardo**

Italy

**Luiz Lopez**

Spain

**Camila Lorenz**

Brazil

**Marcelo Lorenzo**

Brazil

**Vincenzo Lorusso**

France

**Bertrand Losson**

Belgium

**Christos Louis**

Greece

**Van Lun Low**

Malaysia

**Renke Lühken**

Germany

**Julius Lukeš**

Czech Republic

**Zhao-Rong Lun**

China

**Xue-Nong Luo**

China

**Zhiyue Lv**

China

**Issa Lyimo**

Tanzania

**Andrew Macdonald**

United States

**Charles Mackenzie**

United States

**Henry Madsen**

Denmark

**Mathieu Maheu-Giroux**

United Kingdom

**Carla Maia**

Portugal

**Joon Wah Mak**

Malaysia

**Ben Makepeace**

United Kingdom

**Joanna Makol**

Poland

**Arnaldo Maldonado Junior**

Brazil

**Dave Malone**

Tanzania

**Sylvie Manguin**

France

**Am Manonmani**

India

**Ben Mans**

South Africa

**Antonio Marco**

United Kingdom

**Diego Marco**

Argentina

**Sebastien Marcombe**

Lao People's Democratic Republic

**Tanguy Marcotty**

South Africa

**Osvaldo Marinotti**

United States

**Michele Maroli**

Italy

**Gisela Marques**

Brazil

**Josue Martínez-de la Puente**

Spain

**Sandra Maruyama**

Brazil

**Santiago Mas-Coma**

Spain

**Daniel Masiga**

Kenya

**Bruno Mathieu**

France

**Vitor Mati**

Brazil

**Greg Matlashewski**

Canada

**Petra Matoušková**

Czech Republic

**Nancy Matowo**

Tanzania

**Nubia Matta**

Colombia

**Simonetta Mattiucci**

Italy

**Aaron Maule**

United Kingdom

**Isabel Mauricio**

Portugal

**Humphrey Mazigo**

Tanzania

**Charles Mbogo**

Kenya

**Moustapha Mbow**

Senegal

**Nicky McCreesh**

United Kingdom

**Bradford McGwire**

United States

**Anna McKee**

United States

**Don McManus**

Australia

**Carol McNair**

United Kingdom

**Samantha McNulty**

United States

**Henry McSorley**

United States

**Hamish McWilliam**

Australia

**Jan Mead**

United States

**Jansen Medeiros**

Brazil

**Matthew Medeiros**

United States

**Graham Medley**

United Kingdom

**Kim Medley**

United States

**Jolyon Medlock**

United Kingdom

**Paula Medone**

Argentina

**Bastiaan Meerburg**

Netherlands

**Rudolf Meier**

Singapore

**Rudy Meiswinkel**

Italy

**Fábio Melo**

Brazil

**Sergio Mendonca**

Brazil

**Vagner Mendonça**

Brazil

**Pedro Mendoza**

Mexico

**Carlos Mendoza-Palmero**

Mexico

**Louisa Messenger**

United Kingdom

**Antonios Michaelakis**

Greece

**Janet Midega**

Kenya

**Eve Miguel**

France

**Andrei Mihalca**

Romania

**Jocelyn Millar**

United States

**Dennis Minchella**

United States

**Kate Mitchell**

United Kingdom

**Luke Mitchell**

United States

**Kalyan Mitra**

India

**David Modrý**

Czech Republic

**Chang Moh Seng**

Malaysia

**Norhayati Moktar**

Malaysia

**Goudarz Molaei**

United States

**Alvaro Molina-Cruz**

United States

**David Molyneux**

United Kingdom

**Dinesh Mondal**

Bangladesh

**Paul Monis**

Australia

**Fernando Monteiro**

Brazil

**Antonio Montresora**

Switzerland

**Sarah Moore**

United Kingdom

**Daryl Moorhead**

United States

**Jonas Morales Filho**

Brazil

**Jorge Morales-Montor**

Mexico

**Serge Morand**

France

**Alessandra Morassutti**

Brazil

**František Moravec**

Czech Republic

**Felipe Moreira**

Brazil

**Eric Morgan**

United Kingdom

**Neil Morley**

United Kingdom

**Renato Mortara**

Brazil

**Rinaldo Mota**

Brazil

**Maysa Motoki**

Brazil

**Karine Mouline**

France

**Adrian Mountford**

United Kingdom

**Sara Moutailler**

France

**Joachim Mueller**

Switzerland

**Dennis Muhanguzi**

Uganda

**Simon Muhumuza**

Uganda

**Grace Mulcahy**

Ireland

**Andargachew Mulu**

Ethiopia

**Jason Mulvenna**

Australia

**Hetron Munang’andu**

Norway

**Givemore Munhenga**

South Africa

**Francesc Muñoz-Muñoz**

Spain

**Cllaudia Munoz-Zanzi**

United States

**Courtney Murdock**

United States

**Antonio Muro**

Spain

**Julie Mustard**

United States

**Yasen Mutafchiev**

Bulgaria

**Karuppiah Muthumani**

United States

**Ephantus J Muturi**

United States

**Joseph Mwangangi**

Kenya

**Clement Mweya**

Tanzania

**Pauline Mwinzi**

Kenya

**Elmarie Myburgh**

United Kingdom

**Peter Myler**

United States

**Mathios Mylonakis**

Greece

**Byoung-Kuk Na**

Republic of Korea

**Johanna Nader**

United Kingdom

**Behzad Nadjm**

Viet Nam

**Jun Nakagawa**

Japan

**Minoru Nakao**

Japan

**Hira Nakhasi**

United States

**Mark Nanyingi**

Kenya

**Farooq Nasar**

United States

**Julieta Nattero**

Argentina

**Santiago Nava**

Argentina

**Ousmane Ndiath**

Central African Republic

**Daniel Neafsey**

United Kingdom

**Peter Nejsum**

Denmark

**Helena Ngowi**

Tanzania

**Gordon Nichols**

United Kingdom

**Pin Nie**

China

**Birgit Nikolay**

United Kingdom

**Kenneth Nilsson**

Sweden

**Alasdair Nisbet**

United Kingdom

**Veeranoot Nissapatorn**

Malaysia

**Sammy Njenga**

Kenya

**Karsten Noeckler**

Germany

**Nivia Nogueira**

Brazil

**Douglas Norris**

United States

**Patricia Nuttall**

United Kingdom

**Philippe Nwane**

Cameroon

**Anna Obiegala**

Germany

**Adrián Ochoa-Leyva**

Mexico

**Eric Ochomo**

Kenya

**Peter Odermatt**

Switzerland

**Katie O'Dwyer**

New Zealand

**Nick Ogden**

Canada

**Maria Ogrzewalska**

Brazil

**Johanna Ohm**

United States

**Oivind Oines**

Norway

**Fredros Okumu**

Tanzania

**Gaetano Oliva**

Italy

**Jader Oliveira**

Brazil

**Jonathan Oliver**

United States

**Annette Olsen**

Denmark

**Peter Olson**

United Kingdom

**Sheila Ons**

Argentina

**Marinda Oosthuizen**

South Africa

**Laor Orshan**

Israel

**Mohammad Oshaghi**

Iran

**Hugo Osorio**

Portugal

**Domenico Otranto**

Italy

**Johnson Ouma**

Tanzania

**Ellis Owusu-Dabo**

Germany

**Richard Oxborough**

United Kingdom

**Krijn Paaijmans**

United States

**Daniel Paape**

United Kingdom

**Kerry Padgett**

United States

**Soumaïla Pagabeleguem**

Burkina Faso

**Nitu Pages**

Spain

**Timothy Paget**

United Kingdom

**Kathleen Palma**

United States

**Weiqing Pan**

China

**Rosario Panadero**

Spain

**Francisco Panzera**

Uruguay

**Barbara Paoletti**

Italy

**Anna Papa**

Greece

**Elias Papadopoulos**

Greece

**Andrea Paparini**

Australia

**Tony Papenfuss**

Australia

**Daniel Paris**

Thailand

**Joong-Ki Park**

Republic of Korea

**Philippe Parola**

France

**Marilyn Parsons**

United States

**Ilaria Pascucci**

Italy

**Lygia Passos**

Brazil

**Christophe Paupy**

France

**Alex Pauvolid-Correa**

Brazil

**Adolfo Paz**

Spain

**Gustavo Paz**

Brazil

**Anna Paziewska**

Netherlands

**Terry Pearson**

Canada

**James Pecor**

United States

**Adrian Pelin**

Canada

**Cedric Pennetier**

Benin

**Pamela Pennington**

Guatemala

**Jose Mauro Peralta**

Brazil

**Felipe Pereira**

Brazil

**Piyumali Perera**

Australia

**Susan Perkins**

United States

**Stefania Perrucci**

Italy

**Felipe Arley Pessoa**

Brazil

**Grasielle Pessoa**

Brazil

**Jody Peters**

Australia

**Jennifer Peterson**

United States

**Andrew Peterson**

United States

**Dusan Petric**

Serbia and Montenegro

**Martin Pfeffer**

Germany

**Romina Piccinali**

Argentina

**Kim Picozzi**

United Kingdom

**Andrew Pike**

United States

**Somchai Pinlaor**

Thailand

**Adriano Pinter**

Brazil

**Sébastien Pion**

France

**Sebastian Pita**

Uruguay

**Robin Plevin**

United Kingdom

**Nicolas Pocquet**

France

**Mikhail Pomaznoy**

Russian Federation

**Alicia Ponte-Sucre**

Venezuela

**Fleur Ponton**

Australia

**Travis Porco**

United States

**Chuck Porter**

United States

**Aleksandar Potkonjak**

Serbia

**Robert Poulin**

New Zealand

**Jeffrey Powell**

United States

**Penny Powell**

United Kingdom

**Ann Powers**

United States

**Edoardo Pozio**

Italy

**Narendran Pradeep Kumar**

India

**David Price**

United States

**Christian Probst**

Brazil

**Yiannis Proestos**

Cyprus

**Zelia Profeta**

Brazil

**Boonhiang Promdonkoy**

Thailand

**Jorian Prudhomme**

France

**Andrea Pugliese**

Italy

**Rachel Pullan**

United Kingdom

**Whitney Qualls**

United States

**Rupert Quinnell**

United Kingdom

**Martha Quinones**

Colombia

**Tariq Qureshi**

United States

**Juan Antonio Raga**

Spain

**Rao Ramakrishna**

United States

**Juan David Ramirez**

Colombia

**Reda Ramzy**

Egypt

**Shiwanthi Ranasinghe**

Australia

**Manoranjan Ranjit**

India

**Renate Ranka**

Latvia

**Hilary Ranson**

United Kingdom

**Didier Raoult**

France

**Vera Rar**

Russian Federation

**Alberto Rascon**

United States

**Norman Ratcliffe**

United Kingdom

**Ramesh Ratnappan**

United States

**Jean Baptiste Rayaisse**

Burkina Faso

**Paul Ready**

United Kingdom

**Jose Manuel Rebelo**

Brazil

**Guy Reeves**

Germany

**Lisa Reimer**

United Kingdom

**Bobby Reiner**

United States

**George Reis**

Brazil

**William Reisen**

United States

**Michael Reiskind**

United States

**Jan Remme**

France

**Magalie René-Martellet**

France

**Filiberto Reyes-Villanueva**

Mexico

**Alexis Ribas**

Belgium

**José Ribeiro**

United States

**Stephanie Richards**

United States

**David Richardson**

United Kingdom

**Jan Richardus**

Netherlands

**Stanley Rider Jr.**

United States

**Michael Riehle**

United States

**Laura Rinaldi**

Italy

**Scott Ritchie**

Australia

**Marta Riutort**

United States

**Myriam Riyad**

Morocco

**Annapaola Rizzoli**

Italy

**Tamalee Roberts**

Australia

**Derrick Robinson**

France

**Mark Robinson**

United Kingdom

**Sara Robledo**

Brazil

**Maria Robles**

Argentina

**Kat Rock**

United Kingdom

**Boris Rodenko**

United Kingdom

**Eduardo Rodrigues**

Brazil

**Vasco Rodrigues**

France

**Mauricio Rodriguez**

Brazil

**Mario Rodríguez**

Mexico

**David Roiz**

France

**Frederico Rojas**

United Kingdom

**Antonieta Rojas De Arias**

Paraguay

**David Rollinson**

United Kingdom

**Patricia Romano**

Argentina

**Roberto Romi**

Italy

**Thomas Romig**

Germany

**Catherine Ronet**

Switzerland

**André Luiz Roque**

Brazil

**Patricia Rosa**

United States

**Joao Rosa**

Brazil

**Hannah Rose**

United Kingdom

**Gabriele Rossi**

Australia

**Luca Rossi**

Italy

**Xavier Roura**

Spain

**Mark Rowland**

United Kingdom

**Syamal Roy**

India

**Tatiana Rozental**

Brazil

**Miguel Rubio Godoy**

Mexico

**Jonathan Rushton**

United Kingdom

**Tanya Russell**

Australia

**Mike Rust**

United States

**Una Ryan**

Australia

**Jae-Sook Ryu**

Republic of Korea

**Mohammad Khalid Saifullah**

India

**Mostafa Saleh**

Egypt

**Kamila Sales**

Brazil

**Maria Anice Sallum**

Brazil

**Oscar Salomon**

Argentina

**Richard Samuels**

Brazil

**Abdallah Samy**

United States

**Ana Sanchez**

Canada

**Huldah Sang**

United Kingdom

**Rosemary Sang**

Kenya

**Maurício Sant'anna**

Brazil

**Pablo Santo-Orihuela**

Argentina

**Jeannie Nascimento dos Santos Corrêa**

Brazil

**Jacenir Santos-Mallet**

Brazil

**Maria Margarida Santos-Silva**

Portugal

**Manolis Saridomichelakis**

Greece

**Mizuki Sasaki**

Japan

**Sreenivasan Sasidharan**

Malaysia

**Paul Saunderson**

United States

**Katherine Sayler**

United States

**Vera Margarete Scarpassa**

Brazil

**Bjoern Schaeffner**

Brazil

**Juliane Schaer**

Germany

**Guenter Schaub**

Germany

**Theo Schetters**

Netherlands

**Gabriele Schoenian**

Germany

**Thomas Scott**

United States

**Faiza Sebti**

Morocco

**Nagila Secundino**

Brazil

**Otto Seppälä**

Switzerland

**Aklilu Seyoum**

United Kingdom

**Renfu Shao**

Australia

**Paresh Sharma**

India

**Andrew Shatrov**

Russian Federation

**Jeffrey Shaw**

Brazil

**David Shayman**

New Zealand

**Sergei Shekhovtsov**

Russian Federation

**Bang Shen**

China

**Bo Shen**

China

**Jilong Shen**

China

**Chien-Ming Shih**

Taiwan Republic of China

**Andrew Shinn**

United Kingdom

**Oliver Shune**

Zambia

**Afzal Siddiqui**

United States

**Chadwick Sikaala**

Tanzania

**Maggy Sikulu**

Australia

**Cornelia Silaghi**

Switzerland

**Richard Silayo**

United Kingdom

**Maria Helena Silva-Filha**

Brazil

**Jose Silveira**

Bouvet Island

**Ricardo Silvestre**

Portugal

**Frederic Simard**

Burkina Faso

**Andrea Šimková**

Czech Republic

**Gustave Simo**

Cameroon

**Fernando Simón**

Spain

**Bob Sinden**

United Kingdom

**Brajendra Singh**

United States

**Sarman Singh**

India

**Om Singh**

India

**Upinder Singh**

United States

**Marianne Sinka**

United Kingdom

**Steven Sinkins**

United Kingdom

**Edward Sinski**

Poland

**Padet Siriyasatien**

Thailand

**James Skelton**

United States

**Jan Slapeta**

Australia

**Hannah Slater**

United Kingdom

**Nico Smit**

South Africa

**Jennifer Smith**

United States

**Joe Smith**

United States

**Ryan Smith**

United States

**Thomas Smith**

Switzerland

**Cairns Smith**

United Kingdom

**Michael Smout**

Australia

**Robert Snow**

United Kingdom

**Daniel Snyder**

United States

**Daniel Sojka**

Czech Republic

**Cheikh Sokhna**

France

**Philippe Solano**

Burkina Faso

**Laia Solano-Gallego**

Spain

**Aldo Solari**

Chile

**Anthony Solomon**

United Kingdom

**Pradya Somboon**

Thailand

**Christina Sommerville**

United Kingdom

**Marcos Sorgine**

Brazil

**Jani Sormunen**

Finland

**Manuel Soto**

Spain

**Eva Spada**

Italy

**Olivier Sparagano**

United Kingdom

**Hein Sprong**

Netherlands

**Chaichontat Sriworarat**

Thailand

**Claire Standley**

United States

**Damien Stark**

Australia

**Lindsay Starkey**

United States

**Philip Starks**

United States

**Cathy Steel**

United States

**Sasa Stefanic**

Switzerland

**Mario Steindel**

Brazil

**Sonja Steinke**

Germany

**Peter Steinmann**

Switzerland

**Alexandr Stekolnikov**

Russian Federation

**Nicole Stephenson**

United States

**Jamie Stevens**

United Kingdom

**Dietmar Steverding**

United Kingdom

**Roger Stich**

United States

**Patricia Stocco**

Brazil

**Krzysztof Stojecki**

Poland

**Beatriz Stolf**

Brazil

**Wilma Stolk**

Netherlands

**Chris Stone**

Switzerland

**Michael Strand**

United States

**Christina Strube**

Germany

**Snorre Stuen**

Norway

**Chunlei Su**

United States

**Carlos Suarez**

Brazil

**Sarala Subbarao**

India

**Vikrant Sudan**

India

**A.B. Sudeep**

India

**Michael Sukhdeo**

United States

**Devi Suman**

United States

**Xun Suo**

China

**Sinnathamby Surendran**

Sri Lanka

**Bernd Sures**

Germany

**Ben Sutherland**

Canada

**C. Simone Sutherland**

Switzerland

**Daniel Swale**

United States

**Subramanian Swaminathan**

India

**Zainulabeuddin Syed**

United States

**Matias Szabo**

Brazil

**Valentina Tagliapietra**

Italy

**Herbie Tanowitz**

United States

**Mark Taylor**

United Kingdom

**Martin Taylor**

United Kingdom

**Louis-Albert Tchuem Tchuente**

Cameroon

**Alan Rodrigues Teixeira Machado**

Brazil

**Rafael Teixeira-Neto**

Brazil

**Sam Telford**

United States

**Lesly Temesvari**

United States

**Tatiana Teodoro**

Brazil

**Olle Terenius**

Sweden

**Luis Ignacio Terrazas**

Mexico

**Saravanan Thangamani**

United States

**Silvana Carvalho Thiengo**

Brazil

**Frédéric Thomas**

France

**Samuel Thumbi**

United Kingdom

**Zhancheng Tian**

China

**Michel Tibayrenc**

Thailand

**Juan Timi**

Argentina

**Swati Tiwari**

India

**Paolo Tizzani**

Italy

**Rafael Toledo**

Spain

**Paul Torgerson**

Switzerland

**Steve Torr**

United Kingdom

**Eduardo Caio Torres-Santos**

Brazil

**Baldwyn Torto**

Kenya

**Pablo Tortosa**

Reunion

**Philip Toye**

Kenya

**Rebecca Traub**

Australia

**Donato Traversa**

Italy

**Bruno Travi**

United States

**Omar Triana**

Colombia

**Frederic Tripet**

United Kingdom

**Michele Trotta**

Italy

**Anastasios Tsaousis**

United Kingdom

**Stephen Kwok-Wing Tsui**

China

**Michael Turell**

United States

**Kevin Tyler**

United Kingdom

**Preethi Udagama**

Sri Lanka

**Gerrit Uilenberg**

France

**Amy Ullmann**

United States

**Saleh Umair**

New Zealand

**Isik Unlu**

United States

**Suryanaryana Upadhyayula**

India

**Joseph Urban**

United States

**Juerg Utzinger**

Switzerland

**Gustavo Valbuena**

United States

**Anayansi Valderrama**

Panama

**Glyn Vale**

Zimbabwe

**Maria Adela Valero**

Spain

**Gustavo Vallejo**

Colombia

**E.Tellervo Valtonen**

Finland

**Andrew Van Den Hurk**

Australia

**Irma Van Die**

Netherlands

**Jaap J. Van Hellemond**

Netherlands

**Vida Van Staden**

South Africa

**Dana Vanlandingham**

United States

**Amelie Vantaux**

Cambodia

**Sophie Vanwambeke**

Belgium

**André Luís Vanzela**

Brazil

**Anne Vardo-Zalik**

United States

**Marie Varloud**

France

**Jefferson Vaughan**

United States

**Alex Vaux**

United Kingdom

**Muriel Vayssier-Taussat**

France

**Gert Venter**

South Africa

**Laia Ventura-Garcia**

Spain

**José Venzal**

Uruguay

**Jozef Vercruysse**

Belgium

**Tine Verdonck**

Belgium

**Guilherme Verocai**

United States

**Fabrizia Veronesi**

Italy

**Paula Vieira**

Brazil

**Elvina Viennet**

Australia

**Manuela Vilhena**

Portugal

**Tatjana Vilibic-Cavlek**

Croatia

**Maxim Vinarski**

Russian Federation

**Parnpen Viriyavejakul**

Thailand

**Christopher Vitek**

United States

**Roberto Vivancos**

United Kingdom

**Pavel Vlach**

Czech Republic

**Petr Volf**

Czech Republic

**Georg Von Samson-Himmelstjerna**

Germany

**John Vontas**

Greece

**Maarten Voordouw**

Switzerland

**Jan Votýpka**

Czech Republic

**Indra Vythilingam**

Malaysia

**Andrea Waeschenbach**

United Kingdom

**Tania Waghorn**

New Zealand

**Abdul Waheed**

United States

**Jitra Waikagul**

Thailand

**Jessica Waite**

United States

**David Walker**

United States

**Anthony Walker**

United Kingdom

**Edward Walker**

United States

**Richard Wall**

United Kingdom

**Cathy Walton**

United Kingdom

**Joao Wanderley**

Brazil

**Hua Wang**

China

**Cheng-Fang Wang**

China

**Chengming Wang**

China

**Lian Chen Wang**

China

**Xuefeng Wang**

China

**Yuan-Zhi Wang**

China

**Zhong-Quan Wang**

China

**Rongjun Wang**

China

**Xiaoyun Wang**

United States

**Samuel Wanji**

Cameroon

**Alon Warburg**

Israel

**Nicola Wardrop**

United Kingdom

**David Warhurst**

United Kingdom

**Robert Waterhouse**

Switzerland

**Norman Waters**

United States

**Scott Weaver**

United States

**Bonnie Webster**

United Kingdom

**David Weetman**

United Kingdom

**Junfei Wei**

United States

**Gary Weil**

United States

**Mylene Weill**

France

**Susan Welburn**

United Kingdom

**Beth Wells**

United Kingdom

**Christopher Whipps**

United States

**Terry Whitworth**

United States

**Steve Whyard**

Canada

**Giovanni Widmer**

United States

**Stephen Wikel**

United States

**Tom Wilke**

Germany

**Richard Wilkerson**

United States

**Andrew Williams**

Germany

**Kim Williamson**

United States

**R Alan Wilson**

United Kingdom

**William Wint**

United Kingdom

**Margareth Wirth**

United States

**Angelina Wójcik-Fatla**

Poland

**Zhongdao Wu**

China

**Xiang-Yun Wu**

China

**Nigel Wyatt**

United Kingdom

**Chao-Ming Xia**

China

**Lihua Xiao**

United States

**Jiannong Xu**

United States

**Min-Jun Xu**

China

**Wenyue Xu**

China

**Xuenan Xuan**

Japan

**Anges Yadouleton**

Benin

**Chao Yan**

China

**Tetsuya Yanagida**

Japan

**Ting-Bao Yang**

China

**Rui Yang**

China

**Yu-Rong Yang**

China

**Ana Yatsuda**

Brazil

**Matthew Yeo**

United Kingdom

**Serge Yerbanga**

Burkina Faso

**Melissa Yoshimizu**

United States

**Hong You**

Australia

**Neil Young**

Australia

**Xue-Jie Yu**

United States

**Antigoni Zacharopoulou**

Greece

**Stefania Zanet**

Italy

**Magdalena Zarowiecki**

United Kingdom

**Elżbieta Żbikowska**

Poland

**Yifan Zhai**

China

**Kai Zhang**

United States

**Qingfeng Zhang**

China

**Houshuang Zhang**

China

**Rui Zhang**

China

**Sufang Zhang**

China

**Yong-Zhen Zhang**

China

**Hongwei Zhang**

United States

**Shaoruo Zhao**

China

**Guang-Hui Zhao**

China

**Yadong Zheng**

China

**Yong-Tang Zheng**

China

**Elyes Zhioua**

Tunisia

**Dong-Hui Zhou**

China

**Xia Zhou**

China

**Xing-Quan Zhu**

China

**Liang Zhu**

China

**Xinping Zhu**

China

**Dorothee Zielke**

Germany

**Annetta Zintl**

Ireland

